# Anomaly Detection Method for Hydropower Units Based on KSQDC-ADEAD Under Complex Operating Conditions

**DOI:** 10.3390/s25134093

**Published:** 2025-06-30

**Authors:** Tongqiang Yi, Xiaowu Zhao, Yongjie Shi, Xiangnan Jing, Wenyang Lei, Jiang Guo, Yang Meng, Zhengyu Zhang

**Affiliations:** 1 Key Laboratory of Hydraulic Machinery Transients, Ministry of Education, Wuhan University, Wuhan 430072, China; tongqiang.yi@whu.edu.cn (T.Y.);; 2 School of Power and Mechanical Engineering, Wuhan University, Wuhan 430072, China; 3 Shanghai Taishi Education Technology Co., Ltd., Shanghai 430072, China; 4 Key Laboratory of Ecological Safety and Sustainable Development in Arid Lands, Northwest Institute of Eco-Environment and Resources, Chinese Academy of Sciences, Lanzhou 730000, China; 5 School of Materials Science and Engineering, Shanghai University, Shanghai 200444, China

**Keywords:** hydropower units, anomaly detection, operating condition recognition, ensemble learning, density adaptation

## Abstract

The safe and stable operation of hydropower units, as core equipment in clean energy systems, is crucial for power system security. However, anomaly detection under complex operating conditions remains a technical challenge in this field. This paper proposes a hydropower unit anomaly detection method based on K-means seeded quadratic discriminant clustering and an adaptive density-aware ensemble anomaly detection algorithm (KSQDC-ADEAD). The method first employs the KSQDC algorithm to identify different operating conditions of hydropower units. By combining K-means clustering’s initial partitioning capability with quadratic discriminant analysis’s nonlinear decision boundary construction ability, it achieves the high-precision identification of complex nonlinear condition boundaries. Then, an ADEAD algorithm is designed, which incorporates local density information and improves anomaly detection accuracy and stability through multi-model ensemble and density-adaptive strategies. Validation experiments using 14-month actual operational data from a 550 MW unit at a hydropower station in Southwest China show that the KSQDC algorithm achieves a silhouette coefficient of 0.64 in condition recognition, significantly outperforming traditional methods, and the KSQDC-ADEAD algorithm achieves comprehensive scores of 0.30, 0.34, and 0.23 for anomaly detection at three key monitoring points, effectively improving the accuracy and reliability of anomaly detection. This method provides a systematic technical solution for hydropower unit condition monitoring and predictive maintenance.

## 1. Introduction

With the advancement of global energy transformation and carbon neutrality goals, hydropower units, as core equipment in clean energy systems, require safe and stable operation that holds strategic significance for power system security. However, these units feature complex structures and are influenced by multiple factors including hydraulic, mechanical, and electrical elements, making the rapid and accurate identification of operational anomalies under complex operating conditions a major technical challenge for the power industry. Traditional vibration diagnostic techniques exhibit limitations in hydroelectric turbine anomaly detection, including slow processing speeds and narrow frequency measurement ranges [1]. More critically, the substantial variations in normal operational characteristics across different operating conditions further complicate anomaly detection. While advancements in data acquisition technologies have established a foundation for condition assessment through extensive monitoring data, effectively identifying anomalies within these data presents new challenges.

The primary objective of anomaly detection is to identify outlier samples typically resulting from errors, noise, or abnormal behavior [2]. In the field of hydropower unit monitoring, traditional approaches primarily rely on statistical methods, employing techniques such as mean-variance analysis, the 3σ rule, and box plots for anomaly determination [3]. Wu et al. conducted a comprehensive analysis of anomaly identification and data cleaning methods for wind farm power data, highlighting the significant limitations of statistical methods when processing time-series data [4]. Despite their simplicity and intuitive nature, statistical methods face increasingly evident performance bottlenecks as industrial data complexity increases, with their primary constraint being insufficient consideration of internal data structures and temporal correlations. Shen et al. investigated wind–speed–power data distribution characteristics in wind turbines, employing change-point grouping to identify accumulation-type anomalies and quartile methods to eliminate dispersion-type anomalies [5]. Song et al. designed an anomaly detection model combining K-means and random forest for big data, effectively reducing computation time and false positive rates [6]. Both Wang et al.’s Copula function-based wind power anomaly identification model [7] and Hu et al.’s confidence equivalent boundary model [8] demonstrate that when processing complex operational condition data, statistical methods struggle to balance detection sensitivity and accuracy, particularly when temporal correlations exist, where fixed-threshold cleaning strategies often lead to under-deletion or over-deletion, diminishing anomaly detection effectiveness.

With their powerful feature learning and pattern recognition capabilities, machine learning methods demonstrate significant advantages in hydroelectric unit anomaly detection. Mo et al. proposed an isolation forest-based anomaly noise detection method that achieved 97.45% accuracy in hydroelectric unit detection by extracting high-dimensional time-frequency features and determining optimal partition paths [9]. Wang et al. innovatively integrated sliding windows with interval coverage rates to construct a dynamic interval anomaly detection framework, effectively addressing the limitations of point prediction methods [10]. Compared to traditional statistical approaches, machine learning methods adaptively learn complex patterns from data and capture subtle features of anomalies, performing exceptionally well in scenarios with high-dimensional and complex distributions. Guha et al. proposed a robust random partition method based on isolation forests, termed robust random cut forest (RRCF), which improved algorithm robustness against noise by enhancing the isolation forest’s random partition strategy [11]. Chen et al. applied a density and grid-based clustering method to anomaly detection in power monitoring data, achieving favorable identification effects with low false alarm rates [12]. These studies collectively demonstrate that machine learning methods possess unparalleled advantages over traditional approaches in handling complex data distributions and capturing nonlinear anomaly patterns. Zhang et al. employed RRCF for anomaly detection in transformer loss data, demon-strating the method’s computational efficiency and accuracy [13]. Yassin et al. proposed a hybrid detection method combining K-means and Bayesian classifiers, achieving a 95.4% detection rate on the ISCX2012 dataset through a process of clustering followed by identi-fication [14].

However, in actual hydroelectric unit operating environments, anomaly detection methods still face challenges such as variable operating conditions and poor data quality. Hydroelectric unit monitoring data is highly correlated with operating conditions, with significant differences in normal state characteristics under different conditions [15]. Duan et al. addressed issues of highly condition-dependent monitoring data with anomalies and missing values by proposing a condition indicator construction method for hydroelectric units under variable operating conditions based on low-quality data, effectively cleaning low-quality data using the DBSCAN algorithm [16]. Condition-aware anomaly detection strategies are becoming crucial for resolving anomaly identification in multi-condition environments; by first identifying operating conditions and then designing specific detection strategies for different conditions, detection accuracy can be significantly improved while reducing false alarm rates. Tan et al. innovatively combined KD-Tree and DBSCAN technologies to achieve efficient cleaning of hydroelectric unit monitoring data with a correct identification rate of 97.78% [17]. Du et al. proposed a least squares support vector machine-based anomaly monitoring and alarm method for hydroelectric unit equipment to address high false alarm rates in traditional methods, effectively improving monitoring and alarm effectiveness by extracting vibration signal features [18]. These studies collectively indicate that considering condition information is key to improving hydroelectric unit anomaly detection accuracy, and that single anomaly detection models struggle to address the complexity of multi-condition operation.

Based on the above analysis, this paper proposes a hydropower unit anomaly detection method based on KSQDC-ADEAD under complex operating conditions. The method first employs the KSQDC clustering algorithm to identify different operating conditions of hydroelectric units, then designs an improved isolation forest algorithm for anomaly detection according to the characteristics of each condition. The main innovations of this method include the following:

(1) KSQDC Condition Identification Algorithm: A novel two-stage learning paradigm that synergistically combines K-means clustering’s initialization capability with quadratic discriminant analysis’s nonlinear boundary construction. This integration enables precise identification of complex nonlinear condition boundaries and accurate classification of hydroelectric unit operational states.
(2) ADEAD Ensemble Anomaly Detection Algorithm: An adaptive density-aware ensemble framework comprising five complementary sub-models: base isolation forest, extended isolation forest, local density estimation, local outlier factor, and cluster-based scoring. The algorithm enhances detection accuracy and robustness through dynamic inter-model collaboration and adaptive weight fusion strategies.
(3) Integrated Condition-Adaptive Framework: A comprehensive end-to-end system that automates the entire pipeline from data preprocessing and condition identification to anomaly detection. The framework achieves performance optimization through feedback mechanisms and provides a systematic solution for hydroelectric unit health monitoring and predictive maintenance.

This research offers a novel technical approach for hydroelectric unit condition monitoring, effectively improving the accuracy and reliability of anomaly detection and providing robust support for the safe and stable operation of hydropower units, which holds significant importance for advancing clean energy technology development.

The paper is structured as follows: Section 2 reviews related works, including K-means, quadratic discriminant clustering, and isolation forest; Section 3 presents the proposed KSQDC-ADEAD integrated method; Section 4 conducts experimental studies, including data description, experimental design, and result analysis; and Section 5 concludes the research and discusses future work.

## 2. Related Works

### 2.1. K-Means

K-means clustering is one of the most fundamental and widely used unsupervised learning algorithms in data mining and pattern recognition. The algorithm partitions *n* observations into *k* clusters, where each observation belongs to the cluster with the nearest mean, serving as a prototype of the cluster. The standard algorithm was first proposed by Stuart Lloyd in 1957 as a technique for pulse-code modulation, though it was not published until 1982, and recent improvements have focused on initialization and scalability [19]. The algorithm operates by iteratively assigning data points to the nearest centroid and then updating the centroids based on the new cluster assignments [20]. Mathematically, K-means aims to minimize the within-cluster sum of squares (WCSS), also known as the inertia, which is defined as follows:

(1) $$J=\overset{k}{\underset{j=1}{\sum }}\underset{i\in S_{j}}{\sum }\|x_{i}-\mu_{j}{\|}^{2}$$

where $x_{i}$ is the i-th data point, $\mu_{j}$ is the centroid of cluster $S_{j}$, and $\|x_{i}-\mu_{j}{\|}^{2}$ represents the squared Euclidean distance between the data point and the centroid.

However, the K-means algorithm has obvious limitations in practical applications, as its linear decision boundaries based on Euclidean distance struggle to handle complex non-convex data distributions, and it is sensitive to initial centroid selection, easily falling into local optima.

### 2.2. Quadratic Discriminant Clustering (QDC)

QDC looks beyond linear boundaries by leveraging principles from quadratic discriminant analysis (QDA) for clustering tasks. QDC accommodates clusters with different shapes, orientations, and volumes by modeling each cluster using a multivariate Gaussian distribution with its covariance matrix. This provides significantly more flexibility in capturing complex data structures. The mathematical foundation of QDC lies in the probability density function of a multivariate Gaussian distribution:

(2) $$p\left(x|\mu_{k},\Sigma_{k}\right)=\frac{1}{{\left(2\pi \right)}^{d/2}{\left|\Sigma_{k}\right|}^{1/2}}\exp\;\left(-\frac{1}{2}{\left(x-\mu_{k}\right)}^{T}\Sigma_{k}^{-1}\left(x-\mu_{k}\right)\right)$$

where $\mu_{k}$ is the mean vector, $\Sigma_{k}$ is the covariance matrix for cluster *k*, *d* is the dimensionality of the data, and $\left|\Sigma_{k}\right|$ is the determinant of the covariance matrix. The clustering assignment is determined by the quadratic discriminant function:

(3) $$\delta_{k}\left(x\right)=-\frac{1}{2}\log\;\left|\Sigma_{k}\right|-\frac{1}{2}{\left(x-\mu_{k}\right)}^{T}\Sigma_{k}^{-1}\left(x-\mu_{k}\right)+\log\;\pi_{k}$$

where $\pi_{k}$ represents the prior probability of cluster *k*. QDC is implemented through an expectation-maximization (EM) algorithm, which alternates between assigning points to clusters based on the current parameters (E-step) and updating the parameters to maximize the likelihood of the observed data given the assignments (M-step) [21].

Although QDC can construct nonlinear decision boundaries, this method requires the assumption that data follows multivariate Gaussian distributions, and covariance matrix estimation tends to be numerically unstable in high-dimensional data and small sample scenarios. Additionally, QDC lacks an effective mechanism for automatic cluster number determination.

### 2.3. Isolation Forest

**Figure 1:**
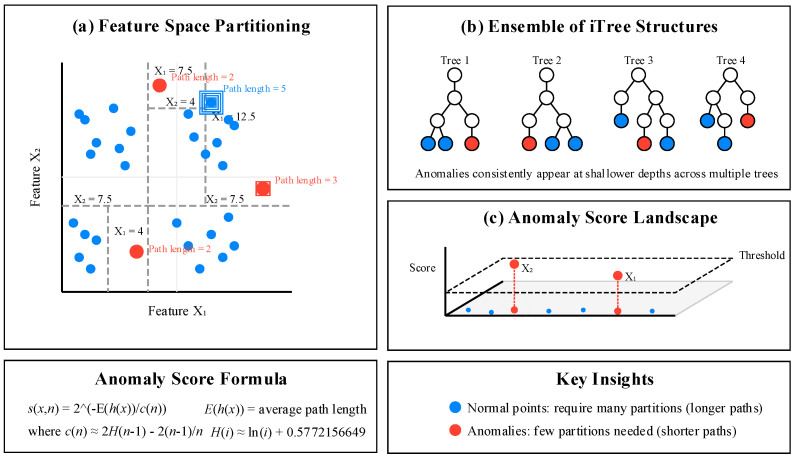
Isolation forest algorithm for anomaly detection.

Isolation forest stands as a revolutionary anomaly detection algorithm that fundamentally differs from traditional density or distance-based approaches. Developed by Liu, Ting, and Zhou in 2008 [22], this method is based on the key insight that anomalies are few and different, making them more susceptible to isolation through random partitioning. Unlike clustering methods that focus on finding similarities among normal data points, isolation forest explicitly isolates anomalies by leveraging the structural characteristics of outliers [23]. The core principle operates through an ensemble of isolation trees, where each tree randomly partitions the data space using a binary tree structure. The algorithm randomly selects a feature and a split value between the maximum and minimum values of that feature, recursively partitioning the data until instances are isolated. Mathematically, the anomaly score for an instance *x* is defined as

(4) $$s\left(x,n\right)=2^{-\frac{E\left(h\left(x\right)\right)}{c\left(n\right)}}\;$$

where E(h(x)) is the average path length for instance *x* across all trees in the forest, and c(n) is the average path length of unsuccessful search in a binary search tree, approximated as

(5) $$c\left(n\right)=2H\left(n-1\right)-\frac{2\left(n-1\right)}{n}$$

with H(i) being the harmonic number estimated as H(i)≈ln (i)+0.5772156649 (Euler’s constant). As illustrated in Figure 1, anomalies (represented by red points) require fewer partitions to be isolated compared to normal points (shown in blue), resulting in shorter path lengths in the isolation trees. The figure demonstrates how the algorithm constructs multiple trees that randomly split the data space, with anomalies consistently appearing in the shallower branches across the ensemble. This visualization highlights the intuitive nature of the method—anomalies are easier to separate from the rest of the data. The three-dimensional plot in Figure 1b further shows how the anomaly scores form a landscape where outliers create distinct peaks, making them easily identifiable through simple thresholding techniques.

While isolation forest performs excellently in anomaly detection, this method has limitations when processing data with significant density variations, and single models struggle to adapt to complex and variable operating conditions. In practical engineering applications, isolation forest is susceptible to changes in data distribution characteristics, leading to unstable detection performance.

## 3. The Proposed Method

### 3.1. KSQDC-ADEAD Integrated Anomaly Detection Method

**Figure 2:**
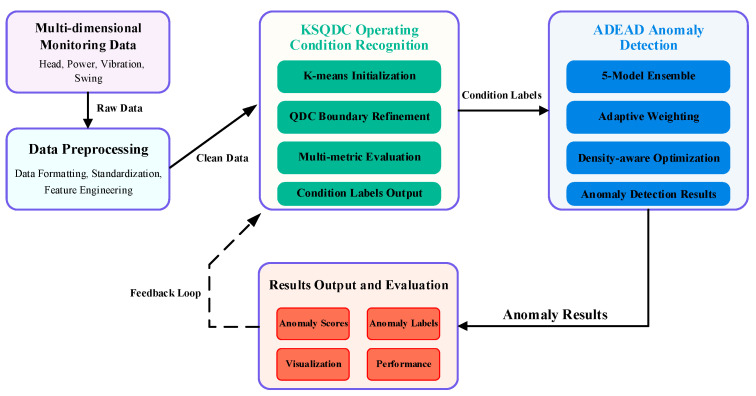
KSQDC-ADEAD overall framework.

The proposed KSQDC-ADEAD method addresses anomaly detection challenges in hydropower units under complex operating conditions through a condition-adaptive strategy. As shown in Figure 2, the method constructs a four-layer progressive architecture, consisting of a data preprocessing layer, a condition recognition layer, a condition-adaptive anomaly detection layer, and a result output layer, achieving end-to-end automated processing from raw multi-dimensional monitoring data to precise anomaly identification.

The core philosophy decomposes the hydropower unit anomaly detection problem into two interconnected sub-problems: operating condition recognition and conditioned anomaly detection. The KSQDC algorithm employs the multi-metric ensemble evaluation mechanism (Section 3.2.2) to automatically determine the optimal number of operating conditions, then identifies unit operating conditions based on head and power features and, subsequently, constructing ADEAD anomaly detection models for each condition, ultimately achieving overall performance optimization through feedback mechanisms.

The methodological implementation follows a systematic five-step execution framework, where each phase builds upon the previous one to ensure comprehensive anomaly detection capabilities:

Step 1: multi-dimensional monitoring data input, encompassing time-series data including water head, active power, vibration, and shaft swing measurements from comprehensive monitoring systems.

Step 2: The data preprocessing module performs basic data formatting, standardization, and feature engineering operations to ensure data consistency and compatibility. This phase implements timestamp synchronization, data format unification, and feature scaling techniques to prepare the dataset for subsequent analysis while preserving all original data patterns.

Step 3: The KSQDC condition recognition phase first applies the four-metric ensemble evaluation (silhouette coefficient, Calinski–Harabasz index, Davies–Bouldin index, and elbow method) to determine the optimal number of conditions, then establishes basic condition partitioning through K-means initial clustering and, subsequently, constructs nonlinear decision boundaries using QDC to achieve precise condition identification.

Step 4: The ADEAD anomaly detection phase constructs five-sub-model ensemble detectors within each identified condition, incorporating base isolation forest, extended isolation forest, local density estimation, local outlier factor, and cluster-based scoring mechanisms. The phase enhances detection precision through adaptive weight fusion and density-aware optimization strategies that dynamically adjust model contributions based on local data characteristics.

Step 5: results output and evaluation, generating anomaly scores, anomaly labels, visualization results, and performance evaluation metrics.

Based on the theoretical analysis above, this section presents the specific implementation procedure of the KSQDC-ADEAD integrated algorithm as detailed in Algorithm 1. The algorithm organically combines operating condition recognition and anomaly detection to achieve end-to-end automated processing. The KSQDC-ADEAD method addresses the growing need for explainable AI in industrial applications by providing inherent interpretability through physically meaningful operating condition recognition and transparent ensemble decision-making. The KSQDC algorithm identifies conditions based on interpretable hydraulic parameters (water head and active power), enabling operators to understand the physical basis of condition assignments. The ADEAD ensemble structure allows tracing anomaly decisions back to individual model contributions, providing transparency about which detection principles (isolation, density deviation, or clustering) drive the final anomaly classification. This interpretability feature is crucial for gaining operator trust and meeting regulatory requirements in critical infrastructure monitoring.


**Algorithm 1: KSQDC-ADEAD integrated framework**

**Input:**
Multi-dimensional monitoring data $x\in R^{d}$, maximum clusters $K_{\max\;}$

**Output:**
Operating condition labels $z_{i}$, anomaly scores $s_{\mathrm{final}\;}\left(x\right)$, anomaly labels *A*
1.
Data preprocessing: $x_{\mathrm{norm}\;}=$ StandardScaler(x)
2.
For K=2 to $K_{\max}$ do
3.
    Compute multi-metric evaluation: $SC_{K},CH_{K},DB_{K},SSE_{K}$

4.
End for
5.
Optimal cluster determination: $K^{*}=\arg\;\underset{K}{\max}\;\mathrm{Voting}\left(SC_{K},CH_{K},DB_{K},SSE_{K}\right)$

6.
K-means initialization: $U_{KM}=\sum_{k=1}^{K}\sum_{i:z_{i}=k}{\left|x_{i}-\mu_{k}\right|}^{2}$

7.
For k=1 to $K^{*}$ do
8.


$\;\;\;\;\mu_{k}=\frac{1}{n_{k}}\sum_{i:z_{i}-k}x_{i}$


9.


$\;\;\;\;\Sigma_{k}=\frac{1}{n_{k}-1}\sum_{i:z_{i}-k}\left(x_{i}-\mu_{k}\right){\left(x_{i}-\mu_{k}\right)}^{T}$


10.


$\;\;\;\;\pi_{k}=\frac{n_{k}}{n}$


11.
End for
12.
QDA boundary refinement: For i=1 to n do
13.


$\;\;\;\;\delta_{k}\left(x_{i}\right)=-\frac{1}{2}\log\Sigma_{k}-\frac{1}{2}{\left(x_{i}-\mu_{k}\right)}^{T}\Sigma_{k}^{-1}\left(x_{i}-\mu_{k}\right)+\log\pi_{k}$


14.


$\;\;\;\;z_{i}={\arg\;\max}_{k}\delta_{k}\left(x_{i}\right)$


15.
End for
16.
For $k\in \left\{1,2,\ldots ,K^{*}\right\}$ do
17.


$\;\;\;\;X_{k}=\left\{x_{i}:z_{i}=k\right\}$


18.
    ADEAD sub-model initialization:
19.
$\;\;\;\;M_{1}=$ BaseIsolationForest ($X_{k}$)
20.
$\;\;\;\;M_{2}=$ ExtendedIsolationForest $\left(X_{k}\right)$
21.
$\;\;\;\;M_{3}=$ LocalDensityEstimation $\left(X_{k}\right)$
22.
$\;\;\;\;M_{4}=$ LocalOutlierFactor $\left(X_{k}\right)$
23.
$\;\;\;\;M_{5}=$ ClusterBasedScoring $\left(X_{k}\right)$
24.
    For i=1 to 5 do
25.
$\;\;\;\;\;\;\;\;s_{i}\left(x\right)=M_{i}$. decision function($X_{k}$)
26.


$\;\;\;\;\;\;\;\;{\hat{s}}_{i}\left(x\right)=\frac{s_{i}\left(x\right)-\min\left(s_{i}\right)}{\max\left(s_{i}\right)-\min\left(s_{i}\right)}$


27.
    End for
28.
    Adaptive weight calculation:
29.


$\;\;\;\;\rho_{i}=\frac{1}{4}\sum_{j∤i}\mathrm{corr}\left({\hat{s}}_{i},{\hat{s}}_{j}\right)$


30.


$\;\;\;\;w_{i}=\frac{\rho_{i}}{\sum_{j-1}^{5}\rho_{j}}\cdot \lambda_{i}+\left(1-\lambda_{i}\right)\cdot w_{i}^{\mathrm{default}\;}$


31.
    Score fusion: $s_{\mathrm{final}\;}\left(x\right)=\sum_{i-1}^{5}w_{i}\cdot {\hat{s}}_{i}\left(x\right)$

32.
    Boundary optimization using silhouette coefficient:
33.


$\;\;\;\;{\mathrm{SC}}_{\mathrm{boundary}\;}\left(x\right)=\frac{d_{\mathrm{inter}\;}\left(x\right)-d_{\mathrm{intra}\;}\left(x\right)}{\max\left\{d_{\mathrm{intra}\;}\left(x\right),d_{\mathrm{inter}\;}\left(x\right)\right\}}$


34.
    If $s\left(i\right)<\tau_{a}$ for anomaly or $s\left(i\right)>\tau_{n}$ for normal then exchange labels
35.
    Update $A_{\mathrm{new}\;}=\left(A\setminus A_{\mathrm{bad}\;}\right)\cup N_{\mathrm{good}\;}$

36.
End for
37.
Return Operating condition labels $z_{i}$, anomaly scores $s_{\mathrm{final}\;}\left(x\right)$, anomaly labels A


### 3.2. K-Means Seeded Quadratic Discriminant Clustering (KSQDC)

KSQDC is a clustering algorithm designed for non-convex shapes and variable density distributions. The algorithm organically combines K-means’ initial partitioning capability with QDA’s non-linear decision boundary construction through a two-stage learning paradigm, improving clustering accuracy and interpretability. The KSQDC algorithm first generates initial clustering with linear decision boundaries, then constructs non-linear decision boundaries through QDA, significantly improving clustering results.

#### 3.2.1. Non-Linear Decision Boundary Construction

The core of the KSQDC algorithm is to transform unsupervised clustering results into prior information for supervised learning, enabling the evolution of decision boundaries from linear to non-linear. Assuming data can be represented by feature vector $x\in R^{d}$, its conditional probability density function under class k is

(6) $$p\left(x|k\right)=\frac{1}{{\left(2\pi \right)}^{d/2}{\left|\Sigma_{k}\right|}^{1/2}}\exp\;\left(-\frac{1}{2}{\left(x-\mu_{k}\right)}^{T}\Sigma_{k}^{-1}\left(x-\mu_{k}\right)\right)$$

where $\mu_{k}\in R^{d}$ and $\Sigma_{k}\in R^{d\times d}$ are the mean vector and covariance matrix of class *k*, respectively. KSQDC endows the model with the ability to capture the unique geometric characteristics of each class by introducing regime-specific covariance matrices.

**Figure 3:**
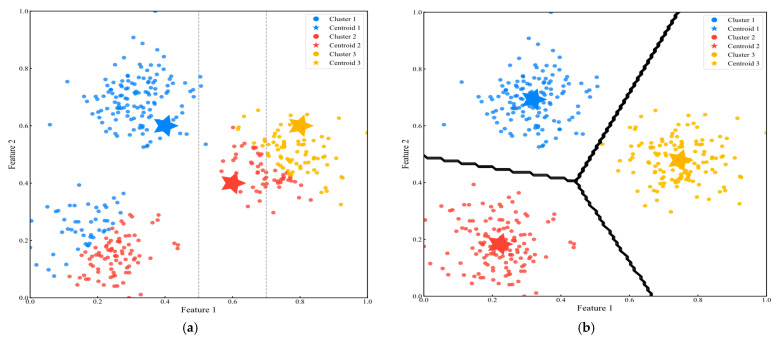
K-means clustering algorithm. ((**a**). Initial state with randomly placed centroids (**b**). Final converged state with optimal centroids and decision boundaries).

The first stage of the algorithm uses K-means to generate initial class labels by minimizing the objective function:

(7) $$J_{KM}=\overset{K}{\underset{k=1}{\sum }}\underset{i:z_{i}=k}{\sum }\|x_{i}-\mu_{k}{\|}^{2}$$

where $z_{i}$ represents the regime label of sample $x_{i}$, and $\mu_{k}$ is the center of the k-th class. Although the initial labels provided by K-means are based on simple Euclidean distance metrics, they provide necessary prior information for the subsequent refined discrimination phase. In Figure 3, the K-means algorithm begins with randomly initialized centroids (represented by stars) and iteratively refines the cluster assignments (shown in different colors) until convergence. The figure illustrates how data points are partitioned into distinct regions based on their proximity to the nearest centroid, forming Voronoi cells in the feature space.

In the refined discrimination phase, QDA constructs non-linear decision boundaries based on Bayesian discrimination theory. For a given sample x, its posterior probability of belonging to class k is

(8) $$P\left(k|x\right)=\frac{\pi_{k}p\left(x|k\right)}{\sum_{j=1}^{K}\pi_{j}p\left(x|j\right)}$$

The discriminant function of QDA is

(9) $$\delta_{k}\left(x\right)=-\frac{1}{2}\log\;\left|\Sigma_{k}\right|-\frac{1}{2}{\left(x-\mu_{k}\right)}^{T}\Sigma_{k}^{-1}\left(x-\mu_{k}\right)+\log\;\pi_{k}$$

where $\pi_{k}$ is the prior probability of regime k. The isosurfaces of the discriminant function form quadratic surface decision boundaries, effectively capturing non-linear boundaries between regimes. For any sample x, its assigned regime label is the class number that $\mathrm{maximizes}\;\delta_{k}\left(x\right)$.

#### 3.2.2. Adaptive Determination of Optimal Number of Classes

A key challenge for the KSQDC method is determining the optimal number of clusters K. This research designs a multi-metric ensemble evaluation mechanism that automatically determines the number of clusters by synthesizing multiple validity indicators. This mechanism balances internal cohesion and external separation and introduces method-specific adjustment factors.

Core evaluation metrics include the silhouette coefficient [24], Calinski–Harabasz Index [25], Davies–Bouldin Index [26], and elbow method [27]. The silhouette coefficient measures the cohesion and separation degree of samples to their assigned classes, calculated as

(10) $$s\left(i\right)=\frac{b\left(i\right)-a\left(i\right)}{\max\left\{a\left(i\right),b\left(i\right)\right\}}$$

where a(i) is the average distance of sample i to other samples in the same class, and b(i) is the average distance of sample i to samples in the nearest different class.

The Calinski–Harabasz Index is based on the ratio of between-class to within-class dispersion, calculated as

(11) $$CH=\frac{\mathrm{tr}\left(B_{K}\right)}{\mathrm{tr}\left(W_{K}\right)}\times \frac{n-K}{K-1}$$

where $B_{K}$ and $W_{K}$ are between-class and within-class dispersion matrices, respectively. The Davies–Bouldin Index measures class compactness and separation, calculated as

(12) $$DB=\frac{1}{K}\overset{K}{\underset{i=1}{\sum }}\underset{j\neq i}{\max}\left\{\frac{\sigma_{i}+\sigma_{j}}{d\left(\mu_{i},\mu_{j}\right)}\right\}\;$$

where $\sigma_{i}$ is the average distance of samples in class i to the class center.

The elbow method determines the optimal number of clusters by calculating the within-cluster sum of squared errors (SSE):

(13) $$SSE=\overset{K}{\underset{k=1}{\sum }}\underset{i\in C_{k}}{\sum }\|x_{i}-\mu_{k}{\|}^{2}$$

where $C_{k}$ represents the k-th cluster and $\mu_{k}$ is the cluster center.

Considering the characteristics of the KSQDC method, a method-specific adjustment factor $\lambda_{\mathrm{KSQDC}}$ is introduced:

(14) $$I_{\mathrm{adjusted}\;}=I_{\mathrm{original}\;}+\lambda_{\mathrm{KSQDC}}\times \frac{K}{K_{\max}}$$

The optimal number of clusters is ultimately determined through a voting mechanism across metrics, where each metric’s optimal *K* value receives one vote, and the K value with the most votes is selected. In case of tied votes, a weighted average determines the final number of clusters.

### 3.3. Adaptive Density-Aware Ensemble Anomaly Detection Algorithm (ADEAD)

**Figure 4:**
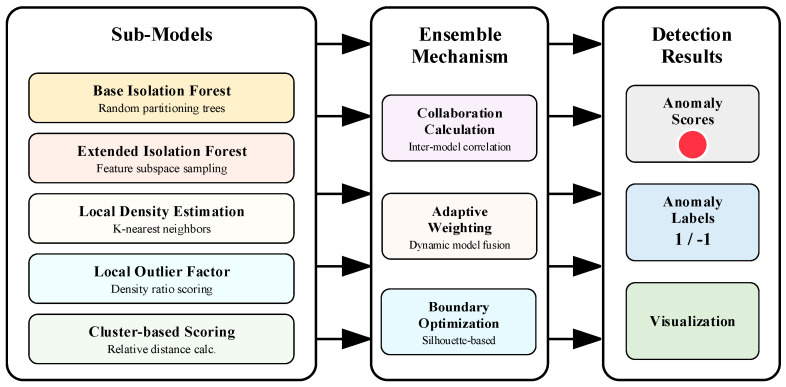
Adaptive density-aware ensemble anomaly detection architecture.

The ADEAD effectively addresses the adaptability limitations of traditional methods to variable density data by integrating multiple complementary anomaly detection sub-models and incorporating local density-sensitive scoring mechanisms. As shown in Figure 4, the ADEAD algorithm consists of five sub-models, uses correlation calculation and adaptive weights for fusion, and then generates final anomaly detection results through boundary optimization.

#### 3.3.1. Multi-Model Ensemble Architecture for Anomaly Detection

The theoretical foundation of the ADEAD method is the combination of ensemble learning and density-aware theory, introducing local density information into anomaly scoring and model fusion processes for the first time. The algorithm constructs five complementary anomaly detection sub-models, each capturing different characteristics of data distribution. The base isolation forest builds isolation trees through random partitioning, evaluating the anomaly degree based on the average path length from samples to leaf nodes; the extended isolation forest adopts an improved random subset sampling strategy for feature space, enhancing sensitivity to anomalies in feature subspaces; the local density estimation provides adaptive local density evaluation based on K-nearest neighbors, enhancing sensitivity to density variations through exponentially weighted distance calculation; the local outlier factor performs anomaly scoring based on the sample local reachability density ratio, effectively identifying anomalies in variable density regions; and the cluster-based scoring combines K-means clustering with relative clustering distance calculation, evaluating the sample dispersion degree relative to its assigned cluster.

For the base isolation forest, the anomaly score $s_{1}$ is calculated as

(15) $$s_{1}\left(x\right)=2^{\frac{E\left(h\left(x\right)\right)}{c\left(n\right)}}$$

where E(h(x)) is the average path length of sample x in the random tree ensemble, and c(n) is a data scale adjustment factor.

For local density estimation, the anomaly score $s_{3}$ is defined as

(16) $$s_{3}\left(x\right)=\frac{1}{k}\overset{k}{\underset{i=1}{\sum }}d\left(x,x_{i}^{NN}\right)\cdot \left(1+\sigma_{d}\left(x\right)\right)$$

where $d\left(x,x_{i}^{NN}\right)$ is the distance from sample x to its i-th nearest neighbor, and $\sigma_{d}\left(x\right)$ is the standard deviation of these distances, introducing variability weighting.

The innovation of the ADEAD method lies in fusing anomaly scores from multiple submodels through an adaptive weighting mechanism:

(17) $$s_{\mathrm{final}\;}\left(x\right)=\overset{5}{\underset{i=1}{\sum }}w_{i}\cdot {\hat{s}}_{i}\left(x\right)$$

where ${\hat{s}}_{i}$ is the normalized anomaly score, and weights $w_{i}$ are dynamically determined through inter-model collaboration:

(18) $$w_{i}=\frac{\rho_{i}}{\sum_{j=1}^{5}\rho_{j}}\cdot \lambda_{i}+\left(1-\lambda_{i}\right)\cdot w_{i}^{\mathrm{default}\;}$$

where $\rho_{i}$ represents the average collaboration degree of the i-th sub-model with other models, $\lambda_{i}$ is a balancing factor, and $w_{i}^{\mathrm{default}\;}$ is the preset base weight.

#### 3.3.2. Density-Adaptive Anomaly Optimization Strategy

**Figure 5:**
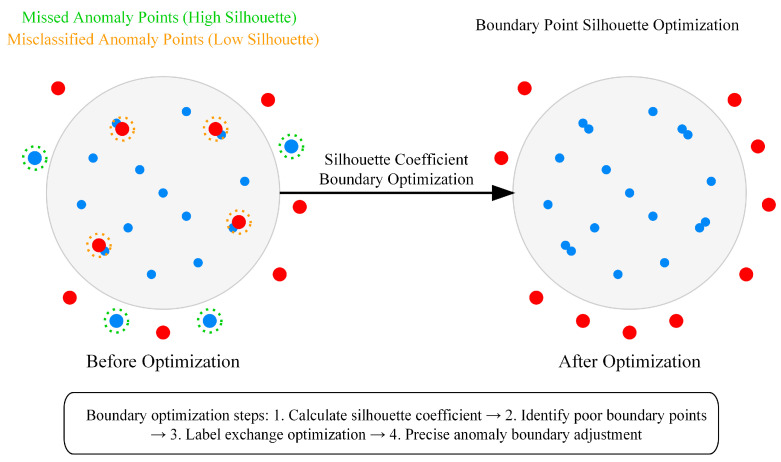
Density adaptive anomaly optimization strategy.

Another key innovation of the ADEAD method is the introduction of a density-adaptive anomaly boundary optimization strategy. As shown in Figure 5, this strategy enables the precise adjustment of anomaly boundaries through silhouette coefficient analysis of boundary samples. For the preliminarily labeled anomaly sample set A and normal sample set N, the boundary optimization silhouette coefficient is calculated as

(19) $${\mathrm{SC}}_{\mathrm{boundary}\;}\left(x\right)=\frac{d_{\mathrm{inter}\;}\left(x\right)-d_{\mathrm{intra}\;}\left(x\right)}{\max\left\{d_{\mathrm{intra}\;}\left(x\right),d_{\mathrm{inter}\;}\left(x\right)\right\}}$$

where $d_{\mathrm{intra}\;}\left(x\right)$ is the average distance from sample x to other samples of the same class, and $d_{\mathrm{inter}\;}\left(x\right)$ is the average distance from sample x to the nearest samples of different classes. The silhouette coefficient provides a score within the range [−1,1] for samples, where a value close to 1 indicates high compatibility with the assigned class, and a value close to −1 suggests possible misclassification.

For anomaly samples $x_{a}\in A$ with lower silhouette coefficients and normal samples $x_{n}\in N$ with higher silhouette coefficients, label exchange is implemented:

(20) $$\begin{array}{r} A_{\mathrm{new}\;}=\left(A\setminus A_{\mathrm{bad}\;}\right)\cup N_{\mathrm{good}\;}\\ N_{\mathrm{new}\;}=\left(N\setminus N_{\mathrm{good}\;}\right)\cup A_{\mathrm{bad}\;} \end{array}$$

where $A_{\mathrm{bad}\;}$ represents the subset of anomaly samples with silhouette coefficients below threshold $\tau_{a}$, and $N_{\mathrm{good}\;}$ represents the subset of normal samples with silhouette coefficients above threshold $\tau_{n}$. The exchange quantity is constrained by maintaining the set anomaly proportion.

## 4. Experimental Study

### 4.1. Data Description

**Figure 6:**
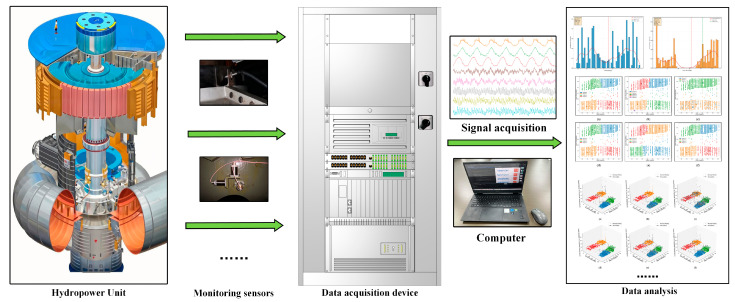
Monitoring and analysis platform for the hydropower unit.

This study utilizes operational data from Unit 4 of a hydropower station in Southwest China for validation, as summarized in Table 1. The unit is a 550 MW mixed-flow turbine with a design head of 165 m and an operating head range of 135–190 m. As illustrated in Figure 6, the hydropower monitoring and analysis platform implements a comprehensive data acquisition and processing architecture. The system is modularly designed and comprises three main stages: signal monitoring, signal acquisition, and signal processing. It encompasses multiple monitoring dimensions—including vibration, shaft displacement, key position, and pressure—to provide real-time, full-scope surveillance of unit operating status. Data were collected at 10-minute intervals over 14 months from July 2023 to September 2024, resulting in a multi-dimensional dataset covering head, power output, vibration, shaft displacement, and other key parameters.

In Table 2, the monitoring system for Unit 4 comprises a comprehensive multi-dimensional network of 31 measurement points across four parameter categories. Water head monitoring is performed at a single sensor, while active power output is recorded in real time by one dedicated power measurement point. Vibration monitoring covers 22 critical locations on the unit—including the lower bracket, stator frame, stator core, upper cover, and upper bracket—with multi-directional accelerometers at each site. Shaft displacement (whirling) is measured bilaterally at the upper guide, water guide bearing, and thrust bearings.

**Figure 7:**
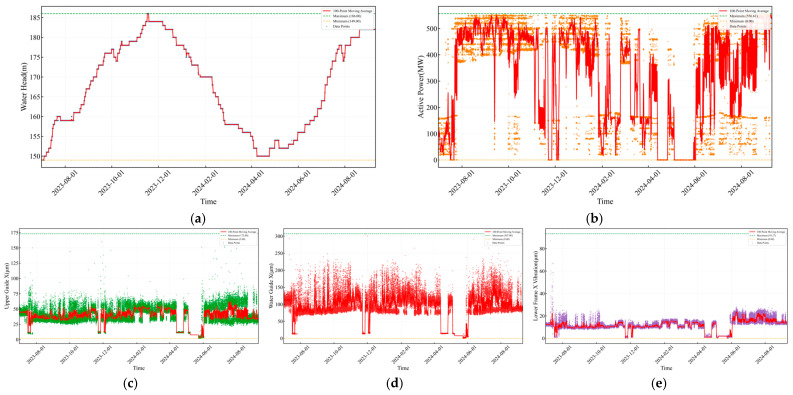
Actual monitoring data of the hydropower unit. (**a**) Water head. (**b**) Active power. (**c**) Upper Guide +X-Axis Swing. (**d**) Water Guide +X-Axis Swing. (**e**) Lower Bracket +X-Axis Vibration.

Figure 7a–e illustrates the temporal evolution of key parameters for Unit 4 during the monitoring period. The head data exhibit pronounced seasonal cycles, with values predominantly ranging from 150 m to 185 m. The overlaid red moving-average line clearly delineates the long-term trend, peaking in May–June 2024—the highest head observed over the entire monitoring window—reflecting both the seasonal inflow patterns of the watershed and upstream reservoir regulation. The active power output demonstrates intermittent operation: It fluctuates between 0 and 500 MW, with frequent zero-power intervals corresponding to unit shutdowns that align with grid-dispatch requirements and underscore the station’s role in peak-load regulation. The Upper Guide + X-Axis Swing remains relatively stable, varying mostly within 0–75 µm; occasional spikes likely correspond to transient conditions during unit startup and shutdown. In contrast, the Water Guide + X-Axis Swing shows greater volatility, concentrating around 50–150 µm under normal operation but occasionally exceeding 175 µm—anomalies that may indicate vortex-rope phenomena or shaft-system instability. Finally, the Lower Bracket + X-Axis Vibration signal displays complex fluctuation patterns: its amplitude correlates with power output and increases markedly during unit startups, shutdowns, and load transitions.

**Figure 8:**
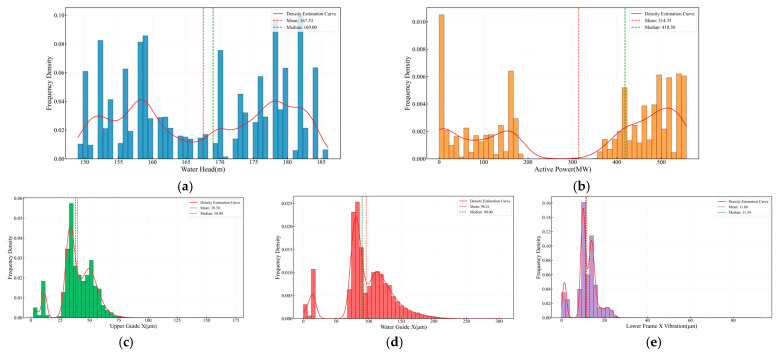
Distribution characteristics of key parameters. (**a**) Water head. (**b**) Active power. (**c**) Upper Guide +X-Axis Swing. (**d**) Water Guide +X-Axis Swing. (**e**) Lower Bracket +X-Axis Vibration.

Figure 8 presents a detailed statistical analysis of each monitored parameter. The head distribution exhibits a bimodal pattern, with a primary mode between 165 m and 175 m and a secondary mode between 155 m and 165 m, reflecting two dominant operating regimes under varying hydrological conditions. The active power output distribution shows three distinct peaks: zero power corresponding to shutdown periods, a mid-range peak at 300–350 MW during normal operation, and a high-power peak at 450–500 MW under full-load conditions. These modes align closely with the operational zones defined in Table 2. The Upper Guide + X-Axis Swing distribution is tightly clustered around 40–60 µm, with a small standard deviation, indicating stable bearing performance. The Water Guide + X-Axis Swing distribution is markedly right-skewed, with a mean of approximately 65 µm and a long upper tail, suggesting intermittent or cyclical displacement anomalies. Finally, the Lower Bracket + X-Axis Vibration distribution is compact and symmetric around 6–12 µm, demonstrating the overall mechanical stability of the unit.

### 4.2. Experimental Design

#### 4.2.1. Experimental Methods

This research adopts a two-stage analytical framework comprising operating condition recognition and anomaly detection. As shown in Table 3, 12 methods were selected for the experiment. In the operating condition recognition stage, six methods (GNB [28], GMM [29], HMM [30], KM [14], HC [31], and KSQDC) were applied to identify the operating conditions of the hydropower unit based on water head (m) and active power (MW). In the anomaly detection stage, six methods (iForest [22], LOF [32], DBSCAN [33], EE [3], OCSVM [34], and ADEAD) were applied to three key monitoring points (Lower Bracket +X Horizontal Vibration, Upper Guide +X Swing, and Water Guide +X Swing) within each identified operating condition. All methods were implemented in Python3.8 using the standard implementations from the scikit-learn library, ensuring fair comparison between methods.

#### 4.2.2. Parameter Configuration

This study optimized algorithm parameters to ensure result reliability. To eliminate randomness effects, all algorithms used a unified random seed (GLOBAL_RANDOM_SEED = 42). Key parameters for condition recognition methods included: the GMM with full covariance matrix and five different initializations, the HMM with 200 maximum iterations, KM with 10 different initial values, original HC using the Ward linkage method for agglomerative clustering, and an improved version using sample size-adapted distance thresholds. The optimal number of conditions was automatically determined through multiple indicators including the silhouette coefficient, CH index, and DB index. Key parameters for anomaly detection methods included the contamination ratio adaptively set based on data size, iForest with 100 trees, the LOF with adaptively adjusted neighbor counts, DBSCAN density threshold calculated through k-distance distribution, the OCSVM boundary parameter set to twice the anomaly ratio, and the ADEAD method with dynamically calculated ensemble weights [0.26, 0.22, 0.20, 0.14, 0.18]. These parameter settings comprehensively considered algorithm stability, computational efficiency, and detection accuracy.

### 4.3. Experimental Results

#### 4.3.1. Analysis of Operating Condition Recognition Results

**Figure 9:**
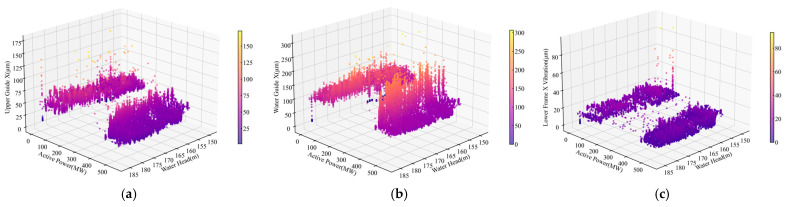
Scatter plots of monitoring signals across all operating conditions. (**a**) Upper Guide +X-Axis Swing. (**b**) Water-Guide +X-Axis Swing. (**c**) Lower Bracket +X-Axis Vibration.

Hydropower units exhibit varying vibration and displacement characteristics under different operating states, making accurate condition recognition a prerequisite for anomaly detection. This section comprehensively evaluates the performance of the proposed method in operating condition identification. As shown in Figure 9, the Upper Guide +X-Axis Swing, Water Guide +X-Axis Swing, and Lower Bracket +X-Axis Vibration exhibit distinct clustering in the head-power-measurement three-dimensional space, with different head and power combinations producing significantly different operating states. The Lower Bracket +X-Axis Vibration (Figure 9c) shows more compact clustering across conditions, indicating higher discrimination capability, while the guide bearing swing measurements demonstrate greater state variability, possibly related to hydraulic disturbances and shaft dynamic characteristics. This discovery provides important clues for subsequent anomaly detection, suggesting different monitoring points have varying diagnostic value.

**Figure 10:**
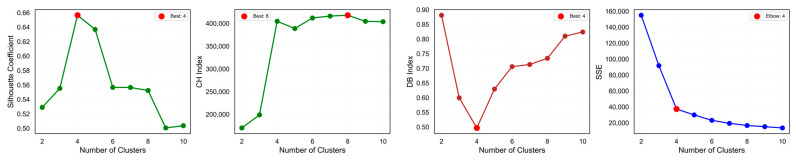
KSQDC operating condition count evaluation results.

Determining the optimal number of operating conditions is a key challenge in clustering analysis. This study employs a multi-metric ensemble evaluation mechanism to address this issue. As shown in Figure 10, we calculated multiple validity indicators including the silhouette coefficient, CH index, DB index, and SSE curve trends as functions of condition count. Results indicate that when the number of conditions is four, the silhouette coefficient (a comprehensive metric measuring cluster compactness and separation) reaches a local maximum, the CH index maintains a relatively high level, the DB index remains relatively low, and the SSE curve shows a clear “elbow point”. This multi-indicator consistency strongly suggests that four is the optimal number of operating conditions. Compared to traditional empirical division or single-indicator assessment, this multi-metric ensemble method significantly improves the scientific validity and reliability of condition recognition.

**Figure 11:**
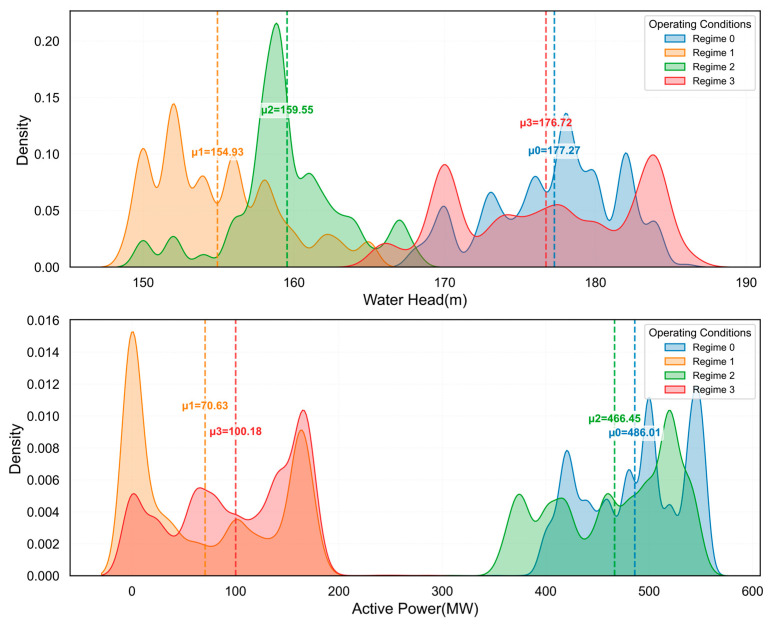
Distribution of operating condition features.

Further analyzing the distribution characteristics of each condition, Figure 11 shows the probability density distributions of four conditions in the water head and active power dimensions. The water head distribution exhibits a clear bimodal characteristic, concentrated in the 155–165 m and 165–175 m intervals, reflecting two primary states of reservoir regulation. Active power presents a more complex multimodal distribution, with shutdown condition (near 0 MW), low load (around 100 MW), medium load (approximately 300 MW), and full load (about 500 MW) as four typical operating ranges, closely related to grid peak-shaving demands. The distribution of conditions in feature space is not simply ellipsoidal but exhibits complex non-convex shapes, which is precisely what traditional linear partitioning methods struggle to effectively handle, and is the primary motivation for the KSQDC method proposed in this paper.

Table 4 further presents the statistical characteristics of each condition quantitatively. Comparing the features of the four conditions reveals that Condition 0 (high water head–high power) accounts for the largest proportion (36.36%), representing the unit’s optimized operation zone; Condition 1 (low water head–low power) follows (26.88%), corresponding to low-load or peak-regulating states; Condition 2 (medium-low water head–high power) represents 20.98%, reflecting power generation dispatch under special hydrological conditions; and Condition 3 (high water head–medium-low power) has the smallest proportion (15.78%), possibly related to ecological flow release under high reservoir levels. From the compactness indicator, Condition 0 shows the highest internal compactness (465.12), indicating the most stable operation, while Condition 3 has the lowest compactness (233.39), displaying greater state fluctuation, possibly related to complex hydraulic conditions during regulation operation under high water head.

#### 4.3.2. Comparison of Different Operating Condition Recognition Methods

**Figure 12:**
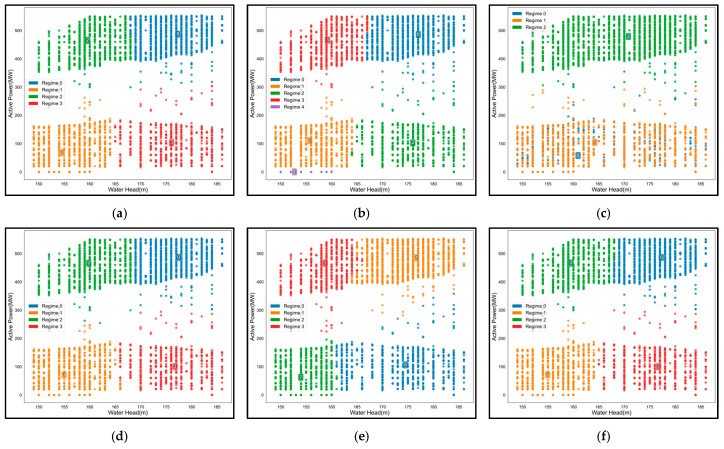
Spatial partitioning effects of six operating condition recognition methods. (**a**) GNB. (**b**) GMM. (**c**) HMM. (**d**) KM. (**e**) HC. (**f**) KSQDC.

To validate the effectiveness of the proposed KSQDC method, we compared several mainstream condition recognition approaches. Figure 12 visually demonstrates the condition recognition results of various methods. From the spatial partitioning results, the methods show different clustering patterns and boundary characteristics. The HMM method (Figure 12c) exhibits considerable overlap between different regions with less distinct boundaries. The GMM method (Figure 12b) identifies five distinct clusters, while most other methods identify four clusters. Traditional clustering methods like K-means (Figure 12d) and HC (Figure 12e) show clear separation between different colored regions. The proposed KSQDC method (Figure 12f) demonstrates well-separated clusters with relatively uniform data point distributions within each cluster.

Table 5 systematically evaluates each method across multiple quantitative indicators. Results show that the KSQDC method delivers the best overall performance on key performance metrics: It achieves the highest silhouette coefficient (SC, 0.64) and condition distribution balance (CDB, 0.98), indicating an optimal balance between internal cohesion and external separation. In terms of computational efficiency, KSQDC’s total execution time is 36.29 s, second only to the HMM’s 36.15 s, ranking second among all methods and demonstrating excellent time efficiency. While the HC method shows slightly higher internal compactness (IC, 384.15 vs. 354.45), its silhouette coefficient (0.60) is lower than that of KSQDC, suggesting that extremely high internal compactness may lead to overfitting; the GMM’s abnormally high internal compactness (71,512.02) further confirms this point, with its overfitting resulting in a notably reduced silhouette coefficient (0.53). It is noteworthy that although the GMM performs relatively well in execution time (37.88 s), its severe performance degradation indicates that computational speed advantages alone cannot compensate for algorithmic limitations. The HMM method performs poorly across all indicators, particularly with a silhouette coefficient of only 0.32, and despite having the shortest execution time (36.15 s), its extremely low recognition accuracy makes it unsuitable for practical engineering applications. These results fully validate the advanced nature and effectiveness of the KSQDC method in addressing nonlinear operating condition recognition problems in hydropower units, achieving the optimal balance between recognition accuracy and computational efficiency.

#### 4.3.3. Analysis of Anomaly Detection Results

**Figure 13:**
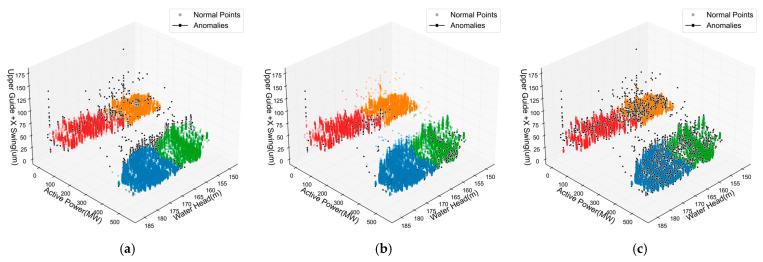

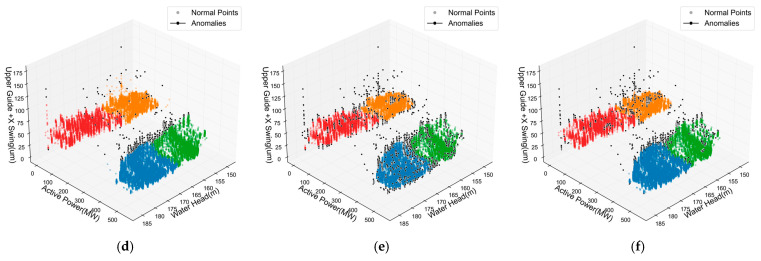
3D scatter plot of anomaly detection results for Upper Guide +X-Axis Swing. (**a**) iForest. (**b**) LOF. (**c**) DBSCAN. (**d**) EE. (**e**) OCSVM. (**f**) ADEAD.

**Figure 14:**
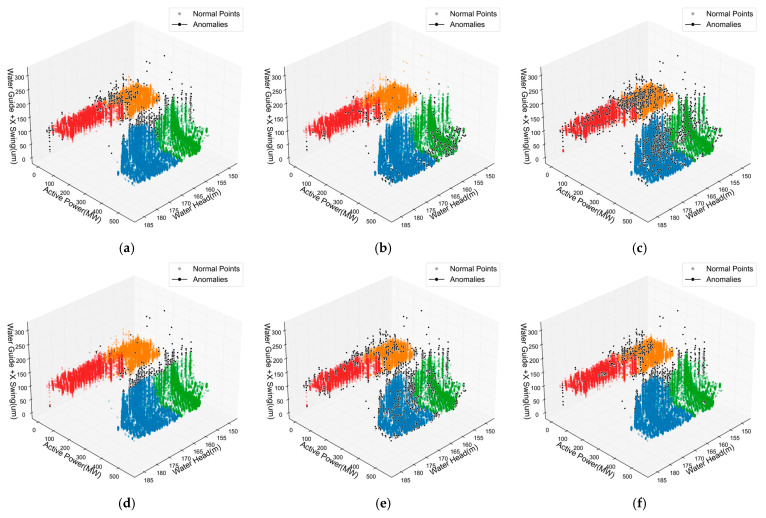
3D scatter plot of anomaly detection results for Water Guide +X-Axis Swing. (**a**) iForest. (**b**) LOF. (**c**) DBSCAN. (**d**) EE. (**e**) OCSVM. (**f**) ADEAD.

**Figure 15:**
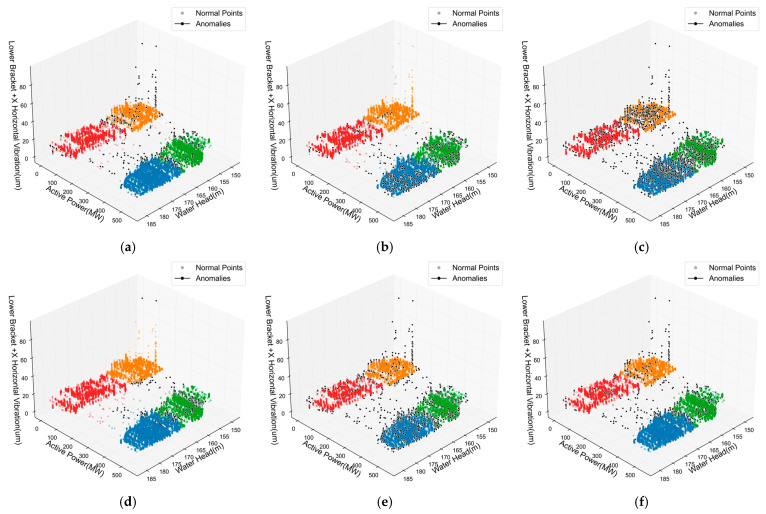
3D scatter plot of anomaly detection results for Lower Bracket +X-Axis Vibration. (**a**) iForest. (**b**) LOF. (**c**) DBSCAN. (**d**) EE. (**e**) OCSVM. (**f**) ADEAD.

From the 3D visualization results shown in Figure 13, Figure 14 and Figure 15, distinct differences in anomaly detection performance across various methods become apparent for the three key monitoring points. The traditional iForest method demonstrates reasonable anomaly identification capabilities but exhibits limitations in boundary precision, particularly in high-density normal sample regions where false positives frequently occur. The LOF method shows advantages in local anomaly detection due to its density-based nature, yet it tends to identify excessive anomaly samples, resulting in higher false alarm rates. DBSCAN’s density clustering characteristics make it effective in low-density regions, but its performance becomes unstable in complex boundary areas where normal and abnormal samples intermingle. In contrast, the proposed ADEAD method demonstrates superior anomaly identification accuracy and stability across all three monitoring points through its multi-model ensemble and density-adaptive strategies, with anomaly sample distributions more closely aligned with the physical principles of actual hydropower unit operations.

The quantitative performance evaluation results presented in Table 6, Table 7 and Table 8 provide comprehensive validation of ADEAD’s superiority. For the Upper Guide +X-Axis Swing monitoring point, ADEAD achieves the highest silhouette coefficient of 0.28 and a comprehensive score of 0.30, significantly outperforming competing methods such as iForest (0.25), the LOF (0.21), and DBSCAN (0.18). In terms of execution time, ADEAD requires a total time of 6.29 s, making it slower than lightweight methods like iForest (0.43 s), the LOF (1.22 s), and DBSCAN (1.37 s), but this time overhead is entirely acceptable considering its superior detection accuracy. The results for Water Guide +X-Axis Swing are even more impressive, with ADEAD reaching a silhouette coefficient of 0.34 and a comprehensive score of 0.34, with a total execution time of 5.60 s, demonstrating enhanced adaptability when processing complex hydraulic disturbance signals. The density ratio metric reveals ADEAD’s balanced approach, achieving 28.33%, 40.22%, and 16.93% for the three monitoring points, respectively, indicating effective adaptation to varying local density characteristics while maintaining consistent anomaly detection accuracy.

The unique anomaly patterns associated with different sensor locations become evident through comparative analysis of detection characteristics across the three monitoring points. Water Guide +X-Axis Swing, as a direct indicator of hydraulic characteristics, presents the highest detection complexity due to vortex rope effects, cavitation, and flow regime transitions. Upper Guide +X-Axis Swing maintains relative stability during normal operation but exhibits transient anomaly features during unit startup and shutdown processes. Lower Bracket +X-Axis Vibration is a comprehensive indicator of overall mechanical condition, with anomaly signals typically related to shaft imbalance, foundation resonance, and other mechanical factors. ADEAD’s incorporation of local density information and adaptive weighting mechanisms enables effective adaptation to these varied anomaly characteristics across different monitoring points, achieving more precise anomaly boundary optimization while completing detection tasks within acceptable time frames, and providing reliable technical support for hydropower unit condition monitoring and predictive maintenance strategies.

This study adopts an unsupervised anomaly detection approach, a choice based on the practical characteristics of hydropower unit operation: Normal operation states occupy the vast majority of time (typically over 95%), while real anomaly events are relatively rare and difficult to pre-label. Unsupervised methods can automatically learn normal operation patterns without relying on extensive labeled data, which better aligns with actual industrial site conditions. To verify the reliability of detection results, we employed multiple validation strategies: First, statistical analysis confirmed that detected anomaly points indeed deviate from normal distribution ranges across monitoring parameters; second, cross-validation with maintenance records and operation logs from the hydropower station revealed a high temporal correlation between detected anomalies and actual equipment faults and maintenance events; and finally, experienced hydropower operation experts were invited to evaluate typical anomaly cases, confirming their engineering reasonableness.

From a physical mechanism perspective, detected anomalies mainly correspond to the following fault modes: Upper guide bearing anomalies primarily manifest as increased axial and radial vibrations, typically caused by bearing wear, poor lubrication, or installation misalignment; water guide bearing anomalies often present as excessive swing, mainly due to hydraulic instabilities causing vortex rope phenomena, cavitation, and abnormal shaft system dynamic responses; and lower bracket vibration anomalies are often related to rotor imbalance, foundation loosening, or transmission system faults. These physical explanations highly align with common fault modes in hydropower units, validating the engineering significance of detection results.

Regarding anomaly processing procedures, detected anomaly points are processed according to severity levels: Minor anomalies are included in trend monitoring with continuous tracking of their development; moderate anomalies trigger warning signals, alerting operators to pay attention and arrange further inspections; and severe anomalies immediately generate alarms, requiring appropriate safety measures. All anomaly information is recorded in the condition monitoring database, providing valuable historical data for subsequent fault diagnosis, maintenance decisions, and model optimization. Meanwhile, anomaly cases confirmed by experts are used to continuously improve the parameter settings and threshold standards of the detection algorithm.

## 5. Conclusions

The proposed hydropower unit anomaly detection method based on KSQDC-ADEAD effectively addresses the challenge of anomaly identification under complex operating conditions. The KSQDC algorithm successfully constructs nonlinear condition decision boundaries by organically combining unsupervised clustering with statistical discriminant analysis, outperforming traditional methods in terms of the silhouette coefficient, internal compactness, and the condition distribution balance, particularly demonstrating superior discrimination capability in condition overlap regions. The ADEAD algorithm significantly improves the accuracy and stability of anomaly detection through multi-model ensemble and density-adaptive optimization strategies, achieving optimal comprehensive scores at all three key monitoring points. The experimental results fully validate the effectiveness and practicality of the proposed method in actual hydropower unit condition monitoring, providing reliable technical support for the safe operation and predictive maintenance of hydropower units.

However, this study also has certain limitations. Compared to recent deep learning methods, this approach has deficiencies in handling large-scale datasets and temporal modeling, as deep learning methods can automatically learn more complex data representations and long-term temporal dependencies. Compared to online learning methods, this approach has limited adaptability to concept drift and requires periodic retraining to cope with long-term changes such as equipment aging. Although this study has made preliminary explorations in interpretability, it has not fully considered deep-level model explanation and causal reasoning mechanisms, which will be an important direction for future research. In ultra-large-scale multi-unit deployments, computational complexity may become a limiting factor. Nevertheless, this method still has obvious advantages in engineering applicability and small-to-medium-scale application scenarios, being particularly suitable for industrial environments with high system transparency requirements.

Future research can be further developed in the following directions: first, combining deep learning technology to construct more complex feature extraction networks to improve the recognition capability of weak anomaly signals; second, introducing temporal information and multi-sensor fusion technology to build a more comprehensive unit health assessment system; third, developing online real-time anomaly detection systems to achieve continuous monitoring and early warning of hydropower unit operating status; and finally, expanding the applicability of the method to apply it to the anomaly detection of other types of rotating machinery equipment, promoting the industrial application and standardized development of related technologies.

## Figures and Tables

**Table 1:** Principal technical specifications of the hydropower unit.

Parameter	Value
Turbine type	Mixed-flow
Rated capacity	550 MW
Design water head	165 m
Operating head range	135–190 m
Rated speed	125 r/min
Commissioning date	Late 1990s

**Table 2:** Monitoring parameter categories and statistics.

Parameter Category	Key Metrics	Measurement Points	Value Range	Sampling Interval
Water pressure	Gross and net head	2	150–185 m	10 min
Power output	Active power	1	0–500 MW	10 min
Vibration	Multi-directional at 22 sites	22	Highly variable	10 min
Shaft displacement	Whirling at guide and thrust bearings (upper guide, lower guide, thrust)	6	0–175 μm	10 min
Total	—	31	—	10 min

**Table 3:** Operating condition recognition and anomaly detection methods.

Stage	Full Method Name	Abbreviation	Fundamental Principle
Operating condition recognition	Gaussian Naive Bayes	GNB	Classification method based on conditional probability and feature independence assumptions
	Gaussian Mixture Model	GMM	Data distribution modeling using weighted combinations of multiple Gaussian distributions
	Hidden Markov Model	HMM	Time-series data analysis through hidden state transition probability modeling
	K-means Clustering	KM	Clustering method minimizing the sum of squared distances from samples to cluster centers
	Hierarchical Clustering	HC	Clustering method that builds a hierarchy of clusters using linkage criteria
	K-means Seeded Quadratic Discriminant Analysis	KSQDC	Hybrid method combining K-means clustering and quadratic discriminant analysis
Anomaly detection	Isolation Forest	iForest	Isolation of sample points through random decision tree construction
	Local Outlier Factor	LOF	Anomaly identification based on local density deviation
	Density-Based Spatial Clustering	DBSCAN	Anomaly detection through identification of high- and low-density regions
	Elliptic Envelope	EE	Identification of distribution edge anomalies by fitting minimum volume ellipsoids
	One-Class Support Vector Machine	OCSVM	Separation of normal samples from anomalies using hyperspheres
	Adaptive Density Ensemble	ADEAD	Integration of multiple basic anomaly detectors with adaptive weight adjustment

**Table 4:** Statistical characteristics and distribution features of four operating conditions.

Operating Condition	Sample Count	Proportion (%)	Water Head Mean (m)	Head Standard Deviation (m)	Active Power Mean (MW)	Active Power Standard Deviation (MW)	Compactness
0	22,191	36.36%	177.29	4.15	486.90	47.55	465.12
1	16,404	26.88%	154.61	3.76	67.84	68.21	426.48
2	12,805	20.98%	159.58	3.81	465.04	57.10	287.53
3	9631	15.78%	176.08	6.31	103.42	59.39	233.39

**Table 5:** Performance metric comparison of various operating condition recognition methods.

Method	IC	CDB	SC	Total Time/s
GNB	353.13	0.96	0.62	38.48
GMM	71,512.02	0.94	0.53	37.88
HMM	239.90	0.89	0.32	36.15
KM	351.48	0.96	0.63	36.58
HC	384.15	0.96	0.60	37.90
KSQDC	354.45	0.98	0.64	36.29

**Table 6:** Performance comparison of anomaly detection methods for Upper Guide +X-Axis Swing.

Method	SC	Anomaly Count	Anomaly Ratio	Density Ratio	Efficiency Score	Separation	Comprehensive_Score	Total Time/s
iForest	0.19	1182	2.00%	17.23%	8.37	2.72	0.25	0.43
LOF	0.11	1171	1.98%	69.37%	21.01	2.48	0.21	1.22
DBSCAN	0.08	1367	2.31%	16.20%	8.87	2.44	0.18	1.37
EE	0.19	1184	2.00%	17.04%	0.44	2.74	0.25	10.95
OCSVM	0.16	2681	4.54%	26.99%	3.44	2.57	0.23	7.39
ADEAD	0.28	1184	2.00%	28.33%	0.88	3.02	0.30	6.29

**Table 7:** Performance comparison of anomaly detection methods for Water Guide +X-Axis Swing.

Method	SC	Anomaly Count	Anomaly Ratio	Density Ratio	Efficiency Score	Separation	Comprehensive_Score	Total Time/s
iForest	0.20	1174	2.00%	23.33%	8.49	2.78	0.26	0.35
LOF	0.12	1155	1.97%	60.49%	20.46	2.47	0.21	1.09
DBSCAN	0.06	1291	2.20%	19.75%	7.37	2.36	0.17	1.27
EE	0.20	1176	2.00%	24.08%	0.45	2.76	0.25	5.87
OCSVM	0.18	2939	5.00%	40.02%	3.88	2.65	0.24	6.02
ADEAD	0.34	1176	2.00%	40.22%	1.09	3.30	0.34	5.60

**Table 8:** Performance comparison of anomaly detection methods for Lower Bracket +X-Axis Vibration.

Method	SC	Anomaly Count	Anomaly Ratio	Density Ratio	Efficiency Score	Separation	Comprehensive_Score	Total Time/s
iForest	0.15	1210	2.00%	17.22%	8.69	2.56	0.22	0.42
LOF	0.10	1208	2.00%	57.77%	18.72	2.40	0.20	1.07
DBSCAN	0.07	1594	2.64%	15.86%	9.82	2.42	0.18	1.34
EE	0.11	1210	2.00%	31.49%	1.13	2.46	0.20	6.42
OCSVM	0.09	2331	3.86%	23.97%	2.79	2.40	0.19	7.32
ADEAD	0.16	1210	2.00%	16.93%	0.43	2.63	0.23	6.75

## Data Availability

The data presented in this study are available on request from the corresponding author due to privacy and confidentiality restrictions.
